# Trouble with Bleeding: Risk Factors for Acute Hepatitis C among HIV-Positive Gay Men from Germany—A Case-Control Study

**DOI:** 10.1371/journal.pone.0017781

**Published:** 2011-03-08

**Authors:** Axel J. Schmidt, Jürgen K. Rockstroh, Martin Vogel, Matthias An der Heiden, Armin Baillot, Ivanka Krznaric, Doris Radun

**Affiliations:** 1 Department for Infectious Diseases Epidemiology, Robert Koch Institute, Berlin, Germany; 2 Medical Clinic I, University of Bonn, Bonn, Germany; 3 Governmental Institute of Public Health of Lower Saxony, Hannover, Germany; 4 Medical Practice, Driesener Strasse, Berlin, Germany; University of Rochester, United States of America

## Abstract

**Objectives:**

To identify risk factors for hepatitis C among HIV-positive men who have sex with men (MSM), focusing on potential sexual, nosocomial, and other non-sexual determinants.

**Background:**

Outbreaks of hepatitis C virus (HCV) infections among HIV-positive MSM have been reported by clinicians in post-industrialized countries since 2000. The sexual acquisition of HCV by gay men who are HIV positive is not, however, fully understood.

**Methods:**

Between 2006 and 2008, a case-control study was embedded into a behavioural survey of MSM in Germany. Cases were HIV-positive and acutely HCV-co-infected, with no history of injection drug use. HIV-positive MSM without known HCV infection, matched for age group, served as controls. The HCV-serostatus of controls was assessed by serological testing of dried blood specimens. Univariable and multivariable regression analyses were used to identify factors independently associated with HCV-co-infection.

**Results:**

34 cases and 67 controls were included. Sex-associated rectal bleeding, receptive fisting and snorting cocaine/amphetamines, combined with group sex, were independently associated with case status. Among cases, surgical interventions overlapped with sex-associated rectal bleeding.

**Conclusions:**

Sexual practices leading to rectal bleeding, and snorting drugs in settings of increased HCV-prevalence are risk factors for acute hepatitis C. We suggest that sharing snorting equipment as well as sharing sexual partners might be modes of sexual transmission. Condoms and gloves may not provide adequate protection if they are contaminated with blood. Public health interventions for HIV-positive gay men should address the role of blood in sexual risk behaviour. Further research is needed into the interplay of proctosurgery and sex-associated rectal bleeding.

## Introduction

Hepatitis C virus (HCV) outbreaks among HIV-positive men who have sex with men (MSM) have been reported by clinicians in post-industrialized countries since 2000 [1], [2]. Outbreaks in this population have been described in Paris [3]–[9], Amsterdam [10], [11], London and Brighton [12], [13], New York City [14], Sydney [15], and in major cities in Switzerland [16] and Germany [17]. Data on HIV serostatus are not routinely collected upon German hepatitis C notification [18], but recent epidemiological surveillance data demonstrated MSM to be the only group showing an increase in diagnosed hepatitis C since 2000 [19]. A large cohort of European HIV-positive MSM who deny injection drug use (IDU) showed a HCV prevalence of 6.6% [20].

Transmission of HCV typically requires direct blood exposure, e.g. through sharing needles or paraphernalia, or through contaminated blood products [21]. HCV has also been identified in body fluids involved in sexual intercourse, such as semen [22]–[25] or vaginal secretions [26], [27], but the mode of sexual transmission of HCV has not yet been determined [16], [28]–[32]. Large longitudinal studies of HCV-serodiscordant heterosexual couples have not yielded significant evidence for sexual transmission; so condom use for the prevention of HCV transmission has not been recommended for vaginal intercourse between monogamous HCV-serodiscordant sexual partners [33]–[36]. Large cohort studies of HIV-negative MSM showed that sexual behaviour, including unprotected anal intercourse (UAI), was not associated with prevalent HCV infections [37]; and those HCV seroconversions rarely observed among MSM without HIV infection could typically be attributed to injection drug use [10], [38]. Certain sexual practices involving trauma of the rectal mucosa have been discussed as relevant risk factors among MSM [13], [39]. Co-infections with bacterial sexually transmitted infections (STIs), especially ulcerative STIs such as syphilis or lymphogranuloma venereum (LGV) have also been proposed as risk factors for HCV transmission among HIV-positive MSM [7], [9], [10], [24], [32], [40]. Furthermore, HIV infection might increase susceptibility towards HCV infection through weakened immunological defence mechanisms [2], [8], [21], [41]. However, there has to date been no attempt to identify the specific mechanisms that, in the context of ‘risky sex’, increase the likelihood of HCV transmission.

Hepatitis C is a major contributor to morbidity and mortality among people living with HIV in post-industrialised countries [42]. Immunosuppression and the HCV genotypes most prevalent among HIV-positive MSM are known to promote treatment failure for HCV-infection [2]. Hepatitis C prevention among HIV-positive MSM is a public health challenge as transmission modes are largely unknown and stigmatization of individuals who are already stigmatized may hamper health-seeking behaviour and disclosure of HCV-seropositivity [43].

The case-control study reported here was conducted to identify risk factors for acute hepatitis C among HIV-positive MSM in Germany, focusing on potential sexual, social, or nosocomial determinants.

## Methods

### Ethical statement

Informed consent was obtained from all participants involved. Because of the anonymity of the questionnaire data, no names and signatures were collected. Approval to having anonymous self-reported data stored and analyzed, and – for controls – to have a dried blood specimen tested for HCV antibodies, was declared by sending back a section of the participant's information paper. Ethic committees of Charité University Clinics in Berlin and of the Medical School of Bonn University approved the study.

### Recruitment of cases and controls

A case-control study was embedded in a national cross-sectional survey of STI knowledge, attitudes and behaviour among German MSM (KABaSTI study [44]). Participants for the case-control study were recruited between September 2006 and January 2008. No incentives were offered to potential participants.

As cases we included HIV-positive MSM who had been diagnosed with acute hepatitis C infection since 2000. They were recruited from an ongoing study of the treatment of acute hepatitis C among HIV-positive individuals at the University of Bonn, where they had been referred from HIV outpatient departments or practice-based physicians specialised in HIV care across Germany. HIV-positive MSM without a history of HCV infection, recruited by the same referring physicians and matched for age groups (±5 years), served as controls. Injection drug use was an exclusion criterion for cases and controls.

### Biological markers

Patients were only chosen as controls if their referring physician, according to patient records, had classified them as HCV-negative. In addition, controls were asked to supply a dried blood specimen (DBS) on 903™ specimen collection paper (Whatman® GmbH, Germany), and to post it inside a multi-barrier pouch, along with the questionnaire, in a prepaid envelope. Capillary blood was collected either by the referring physician or by the participant himself, using an enclosed sterile lancet (Haemostiletten®, ASID BONZ GmbH, Germany), following the instructions provided. The HCV serostatus of controls was confirmed with an automated chemiluminescent microparticle immunoassay (CMIA, ARCHITECT anti-HCV) from DBS [45].

### Questionnaires

The KABaSTI study questionnaire has been described in detail elsewhere [46]. Briefly, an anonymous, 66-item self-completion questionnaire was distributed, covering socio-demographic data; sexual behaviour and diagnoses of STIs in the last 12 months; attitudes towards condom use to prevent transmission of STIs other than HIV, attitudes towards condom use with HIV-seroconcordant sexual partners; and HIV serostatus of non-steady sex partners.

Additionally, we developed a 23-item questionnaire to gather data on specific sexual, nosocomial, and other non-sexual exposures potentially associated with transmission of HCV, focusing on the time since 2000 as all cases included in this study seroconverted after 2000. The time frame for cases thus refers to the time frame between 2000 and hepatitis C diagnosis. Variables are described in table 1 and table 2.

**Table 1:** Socio-demographic data.

Category	Subcategory	Cases (%)	Controls (%)
N		34 (100.0)	67 (100.0)
Age	20–29 years	4 (11.8)	9 (13.4)
	30–44 years	24 (70.6)	48 (71.6)
	45–65 years	6 (17.6)	10 (14.9)
	Mean ± SD	39±8	39±7
	Median (range)	41 (22–65)	41 (20–57)
Age at first sex with a man	Mean ± SD	17±3	17±4
	Median	17	17
Year of HIV diagnosis	Mean ± SD	1999±5	1999±7
	Median (range)	2000 (1987–2007)	2001 (1987–2007)
Self reported last CD4 count	>350/µL	22 (64.7)	44 (65.7)
	200–350/µL	4 (11.8)	13 (19.4)
	<200/µL	2 (5.9)	2 (3.0)
	Unknown	6 (17.6)	8 (11.9)
Antiretroviral therapy	Presently taking	28 (82.4)	50 (74.6)
Screening for HCV	Ever offered	27 (79.4)	54 (80.6)
Year of HCV diagnosis	Mean ± SD	2005±1	—
	Median (range)	2005 (2003–2007)	—
City size	≥500,000	31 (91.2)	50 (74.6)
Education	General qualification for university	17 (50.0)	35 (52.2)
Employment status	Retired/unemployed	13 (41.2)	26 (38.8)
Relationship status	Single	13 (38.2)	26 (38.8)
Sexual identification	Gay	33 (97.1)	67 (100.0)

**Table 2:** Exposures and potential risk factors for acute hepatitis C; bold if included in multivariable analysis.

Category	Subcategory	Cases (%)	Controls (%)	Univariable		Multivariable	
				OR (95%- CI)	p	Adj. OR (95%- CI)	p
N		34 (100.0)	67 (100.0)				
*Sexual behaviour in the previous 12 months*							
**Number of sex partners**	**More than 10**	22 (64.7)	33 (49.3)	1.89 (0.81–4.42)	0.204	—	0.939
	More than 20	17 (50.0)	27 (40.3)	1.48 (0.65–3.40)	0.400	—	0.641
	More than 50	7 (20.6)	13 (19.4)	1.08 (0.39–3.01)	1.000		
Anonymous sex partners	More than 50 per cent	16 (47.1)	30 (44.8)	1.10 (0.48–2.51)	0.836		
Sex frequency	Twice weekly or more	9 (26.5)	17 (25.4)	1.06 (0.41–2.71)	1.000		
Met sex partners through the internet	Often or always	18 (52.9)	26 (38.8)	1.77 (0.77–4.08)	0.206		
Met sex partners in saunas	Often or always	5 (14.7)	9 (13.4)	1.11 (0,34–3.62)	1.000		
Met sex partners at sex parties	Often or always	10 (29.4)	12 (17.9)	1.91 (0.73–5.02)	0.209		
Met sex partners at leather venues	Often or always	8 (23.5)	8 (11.9)	2.27 (0.77–6.70)	0.155		
**Insertive AI** § **with non-steady partners**	**Frequently or always**	21 (61.8)	24 (35.8)	2.89 (1.23–6.79)	0.019	—	0.347
**Receptive AI** § **with non-steady partners**	**Frequently or always**	20 (58.8)	26 (38.8)	2.25 (0.97–5.23)	0.062		0.759
**BDSM** §§ **or fisting with non-steady partners**	**Frequently or always**	10 (29.4)	9 (13.4)	2.69 (0.97–7.44)	0.063	—	0.708
**UAI with non-steady partners** *	**One episode or more**	22 (64.7)	25 (37.3)	3.08 (1.30–7.28)	0.012	—	0.288
	**Five episodes or more**	15 (44.1)	14 (20.9)	2.99 (1.22–7.33)	0.020	—	0.653
*STIs and condom attitudes*							
**History of syphilis**	**In the last 12 months**	10 (29.4)	9 (13.4)	2.69 (0.97–7.44)	0.063		0.192
History of Chlamydia infection	In the last 12 months	7 (20.6)	10 (14.9)	1.48 (0.51–4.30)	0.575		
History of gonorrhoea	In the last 12 months	8 (23.5)	12 (17.9)	1.41 (0.51–3.87)	0.599		
**History of any of the three above**	**In the last 12 months**	19 (55.9)	21 (31.3)	2.78 (1.18–6.50)	0.020	—	0.211
Non-steady partners with syphilis	In the last 12 months	5 (14.7)	10 (14.9)	0.98 (0.31–3.14)	1.000		
**Non-steady partners with HIV**	**Presently**	27 (79.4)	38 (56.7)	2.94 (1.13–7.70)	0.029	—	0.986
Would engage in UAI if sex partner is seroconcordant for HIV		22 (64.7)	39 (58.2)	1.32 (0.56–3.09)	0.667		
**Would never engage in any UAI**		4 (11.8)	22 (32.8)	0.27 (0.09–0.87)	0.029	—	0.296
**Condoms not considered for prevention of STIs (other than HIV)**		14 (41.2)	13 (19.4)	2.91 (1.17–7.24)	0.031	—	0.554
*Sexual exposures since 2000*							
UAI* with person known to be HCV positive		3 (8.8)	5 (7.5)	1.20 (0.27–5.35)	1.000		
**Group sex**	**Yes**	31 (91.2)	44 (65.7)	5.40 (1.49–19.58)	0.007	3,50 (0.84–14.52)	0.084
	**Frequently or always** †	15 (44.1)	14 (20.9)	3.00 (1.22–7.33)	0.020	—	0.786
**PDE-5 inhibitors** **	**Frequently or always** †	15 (44.1)	12 (17.9)	3.62 (1.44–9.09)	0.008	—	0.557
Nitrite inhalants or large amounts of alcohol	Frequently or always†	22 (64.7)	36 (53.7)	1.58 (0.67–3.70)	0.395		
**Dilative sex toys**	**Usage**	27 (79.4)	37 (55.2)	3.13 (1.20–8.17)	0.028	—	0.588
Frequently sharing dilative sex toys		3 (8.8)	2 (3.0)	3.15 (0.50–19.80)	0.332		
**Anal pain with bloody diarrhoea**	**Repeatedly**	6 (17.6)	4 (6.0)	3.38 (0.88–12.91)	0.082	—	0.384
**Rectal trauma with bleeding**	**Frequently or always** †	7 (20.6)	3 (4.5)	5.53 (1.33–23.00)	0.029	6.19 (1.17–32.81)	0.032
Skin damage (BDSM§§) with bleeding	Yes	8 (23.5)	9 (13.4)	1.98 (0.69–5.72)	0.261		
Receptive fisting	Yes	17 (50.0)	15 (22.4)	3.47 (1.43–8.39)	0.007		
	Frequently or always†	12 (35.3)	6 (9.0)	5.55 (1.86–16.57)	0.002		
**Frequent receptive fisting without gloves (or gloves shared)**		11 (32.4)	4 (6.0)	7.53 (2.18–26.03)	0.001	5.71 (1.50–21.68)	0.011
**Frequent receptive fisting and sharing of lubricant**		10 (29.4)	5 (7.5)	5.17 (1.60–16.69)	0.006	—	0.603
*Nosocomial exposures*							
Blood or plasma transfusion	Any before 1991	2 (5.9)	5 (7.5)	0.78 (0.14–4.22)	1.000		
**Major surgery**	**Repeatedly**	11 (32.4)	11 (16.4)	2.44 (0.93–6.40)	0.079	—	0.507
Major dental treatment	Repeatedly	2 (5.9)	10 (14.9)	0.36 (0.07–1.73)	0.329		
Endoscopies (gastro-, colono-, cystoscopies)	Repeatedly	13 (38.2)	23 (34.3)	1.18 (0.50–2.79)	0.826		
Acupuncture	Repeatedly	2 (5.9)	5 (7.5)	0.78 (0.14–4.22)	1.000		
*Other exposures since 2000*							
Tattooing	Repeatedly	4 (11.8)	9 (13.4)	0.86 (0.24–3.02)	0.812		
**Body-piercing**	**Repeatedly**	7 (20.6)	5 (7.5)	3.22 (0.94–11.04)	0.099	—	0.529
Imprisonment	More than 72 h	0 (0.0)	2 (3.0)	—	0.549		
Shared household with HCV positive person		4 (11.8)	5 (7.5)	1.60 (0.40–6.40)	0.489		
**Consumption of NADs** ***	**Yes**	28 (82.4)	35 (52.2)	4.27 (1.56–11.64)	0.004	3,25 (1.06–9.96)	0.040
	**Monthly or weekly**	7 (20.6)	3 (4.5)	5.53 (1.33–23.00)	0.029	—	0.135
**Consumption of NADs and sharing of equipment**		22 (64.7)	25 (37.3)	3.08 (1.30–7.28)	0.012	—	0.804
*Multivariable model summary*							
Hosmer-Lemeshow-Test (χ-square; p)						6.925	0.226
Nagelkerke's pseudo-R-square						0.327	

§ AI = anal intercourse;

§§ BDSM = bondage, dominance/submission, sadism/masochism;

* UAI = unprotected AI with non-steady partners of unknown HIV serostatus;

** PDE-5 = phosphodiesterase-5: The risk of penile (rectal) trauma is higher if intercourse is temporally extended by (the partner's) use of PDE-5 inhibitors;

*** NADs = nasally-administered drugs (cocaine, amphetamines, ketamine, etc.);

† frequently or always when having sex.

### Sample size estimation

We assumed a prevalence of suspected sexual exposures among controls of ≥15%. This is in line with published literature on behavioural surveillance among MSM in Germany (e.g. attending sex parties, snorting cocaine, diagnosed with syphilis, and BDSM [bondage, sadism/masochism] or fisting with non-steady partners) [44], [47]. To detect Odds Ratios of ≥3.0 with a power of 80% and an alpha-level of 5%, we aimed at 53 cases and 159 controls, at a case-control ratio of 1∶3.

### Statistics

Due to expected small numbers, two-sided Fisher's Exact Test was used in univariable analysis to determine if an Odds Ratio (OR) was significantly different from 1. ORs and an approximated 95% confidence interval were calculated with SPSS 16. We looked for confounding factors and effect measure modifiers by stratification. Where effect measure modification was present, we constructed interaction terms that were tested individually for statistical significance.

To identify factors independently associated with acute hepatitis C, variables with ORs significantly different from 1 (p<0.10), were entered into multivariable logistic regression by stepwise forward and backward selection (Wald, cut-off at p<0.10). When effect measure modification was present among the remaining factors, we defined interaction terms that were tested for a statistically significant influence on the outcome parameter. Goodness-of-fit of the models was evaluated with Hosmer-Lemeshow test, and Nagelkerke's pseudo-R-square to estimate the explanatory power of the models [48]. Venn diagrams were used to visualize and to compare overlap of exposures among cases and controls. The circular areas correspond with the respective presence of exposures.

## Results

Overall, 39 men were recruited as cases and 78 as controls. Of the cases, five had to be excluded: three had a history of IDU, one became HCV-infected before 2000, and one provided implausible responses. Of the controls, one was excluded because of IDU; eight because they were not HIV-positive, and two due to inconsistent responses. Thus, 34 cases and 67 controls were included in the univariable and multivariable analyses. Of the controls, 43 (64%) provided DBS. All specimens tested negative for HCV antibodies.

### Socio-demographic characteristics of study population

The median age of respondents was 41 years, ranging from 20 to 65 years. Of the 34 cases, most were living in Berlin (n = 27), Frankfurt (n = 3), Hamburg (n = 1), or small cities in northern and south-western federal states. Of the 67 controls, most were living in Berlin (n = 37), Frankfurt (n = 10), Cologne/Bonn (n = 9), Mannheim (n = 1), the Ruhr (n = 4), or small cities in south-western federal states (n = 6). Cases and controls did not differ with respect to age, education, employment, sexual debut with a man, relationship status, ever being screened for HCV, year of HIV diagnosis, self-reported last CD4 count, or being on antiretroviral treatment. All participants but one self-identified as gay (table 1).

### Univariable analysis: Sexual behaviour and STIs in the last 12 months, and attitudes towards condom use

Some differences were found between cases and controls with respect to sexual behaviour during the twelve months preceding study participation (table 2). More than ten sex partners were reported by 65% of the cases vs. 49% of the controls. Applying higher cut-off values for dichotomisation (more than twenty, or more than fifty sex partners), did not result in a statistically significant association with hepatitis C. Cases reported more anal intercourse with non-steady partners, and were more likely to report episodes of unprotected anal intercourse (UAI) with non-steady partners of unknown HIV serostatus than controls (65% vs. 38%). More cases than controls had non-steady sex partners who were HIV-positive (79% vs. 57%), and reported more diagnoses of syphilis, Chlamydia infection, or gonorrhoea (56% vs. 31%); and also less often considered condoms for STI-prevention (41% vs. 19%).

Among cases as among controls, a majority said they would not use a condom if sex partners were seroconcordant for HIV; but cases were less likely to reject any anal intercourse without a condom (12% vs. 33%).

### Univariable analysis: Specific sexual exposures since 2000

As to the time period since the year 2000, 9% of cases reported UAI with a sex partner known to be HCV positive; so did 8% of controls (table 2). Group sex since the year 2000 was reported by 91% of cases vs. 66% of controls (OR = 5.4); and 44% vs. 21% said that, when having sex, this had been frequently or always in a group of men (table 2).

Anorectal trauma with subsequent visible bleeding was a frequent or regular experience for 21% of the cases (vs. 5% of the controls), showing a strong association with acute hepatitis C (OR = 5.5). Frequent or regular use of phosphodiesterase-5 (PDE-5) inhibitors was reported by 44% of cases vs. 18% of controls.

Receptive fisting was even more common: 50% of the cases vs. 22% of the controls reported receptive fisting since 2000; and 35% vs. 9% said they had been fisted ‘frequently or always when having sex’. Only 6 out of all 32 receptive ‘fisters’ (19%) said their partner(s) had mostly worn gloves (to cover hands and forearms) for fisting. Moreover, 12 out of 17 (71%) HCV/HIV co-infected, and 8 out of 15 (53%) HIV mono-infected receptive ‘fisters’ reported using some sort of collective lubricant supply. Cases had a 7.5 times higher odds of reporting a combination of frequent fisting and not using gloves (or sharing them) than controls did; and a 5.2 times higher odds of reporting a combination of frequent fisting and sharing of lubricant.

### Univariable analysis: Nosocomial and other non-sexual exposures since 2000

We observed no differences between cases and controls regarding most nosocomial exposures. Blood or plasma transfusions before 1991 (when screening of donors for HCV antibodies was introduced in Germany), were as common among cases as among controls, as were multiple exposures to gastroscopies, colonoscopies, cystoscopies, major dental treatment, or acupuncture since the year 2000. Body piercings (21% vs. 8%), but not tattoos, were more common among cases than among controls. More cases than controls reported a history of multiple episodes of major surgery (32% vs. 16%). Surgery was associated with frequent group sex (OR = 5.69; p<0.001), more than twenty sex partners in the previous twelve months (OR = 3.70; p = 0.008), frequent receptive (but not insertive) anal intercourse with non-steady sex partners (OR = 3.32; p = 0.015), and particularly with having HIV-positive non-steady sex partners (OR = 7.56; p = 0.002), but not with fisting or age.

Among non-sexual exposures, consumption of nasally-administered drugs (NADs) – cocaine, amphetamines, or ketamine – was associated with HCV co-infection. More cases than controls reported that they had consumed NADs since the year 2000 (85% vs. 52%), and more had done so monthly or weekly (21% vs. 5%; OR = 5.5). Both groups reported sharing of snorting equipment (79% vs. 71%). A combination of NAD consumption and sharing of related equipment was especially common among cases (65% vs. 37%).

### Multivariable logistic regression analysis

We identified frequent rectal trauma with bleeding, frequent receptive fisting without gloves, group sex, and consumption of NADs as independent risk factors for acute hepatitis C virus infection, with Nagelkerke's pseudo-R-square of 32.7% (table 1). Effect measure modification was present between fisting/bleeding; fisting/consumption of NADs; fisting/group sex; and between group sex/consumption of NADs. By including these interaction terms, consumption of NADs and group sex were replaced by their respective interaction term (OR = 5.91; 95%-CI: 2.04–17.14; p = 0.001). Nagelkerke's pseudo-R-square for the remaining three variables was 34.6%. In 32 out of 34 cases (94%), at least one of these three factors was present. The model retained its robustness when restricting the multivariable analysis to controls with confirmed HCV-negative serostatus.

### Overlap of exposures

**Figure 1:**
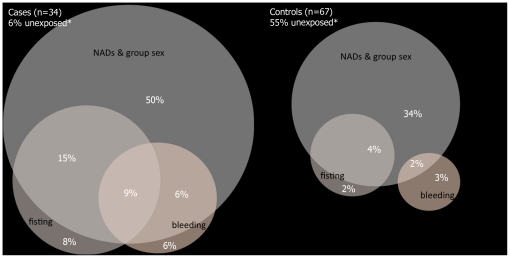
Venn diagram for overlap of exposures (frequent receptive fisting without gloves, frequent anal bleeding, group sex & consumption of NADs). *Unexposed: Respondents who neither had been frequently fisted without gloves (or with gloves that were shared), nor frequently experienced anal bleeding when having sex, nor reported group sex activities plus consumption of NADs. The circular areas correspond with the respective presence of exposures; intersecting areas reflect only roughly the given percentages.

**Figure 2:**
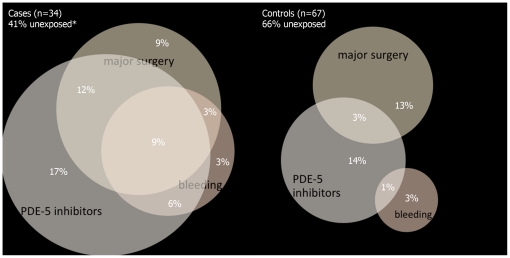
Venn diagram for overlap of exposures (frequent use of PDE-5 inhibitors, frequent anal bleeding, history of major surgery). *Unexposed: Respondents who neither had frequently used PDE-5 inhibitors, nor frequently experienced anal bleeding when having sex, and who did not report multiple surgical interventions. The circular areas correspond with the respective presence of exposures; intersecting areas reflect only roughly the given percentages.

There was an overlap between frequent receptive fisting without gloves and frequent rectal trauma with bleeding among cases (9% reported both exposures), whereas among controls, no such overlap was present (figure 1). All risk factors independently associated with HCV infection showed a higher degree of overlap among cases. Neither fisting nor rectal bleeding was associated with a recent history of bacterial STIs (p = 0.577; p = 0.512), a higher number of sex partners (p = 0.403; p = 1.000), or unprotected anal intercourse with a partner of unknown HIV serostatus (p = 0.278; P = 0.501). Among cases, but not among controls, there was substantial overlap between a history of multiple episodes of major surgery and rectal bleeding, or use of PDE-5-inhibitors (figure 2).

## Discussion

This case-control study adds information on risk factors for acute hepatitis C among gay men with HIV infection. Our findings suggest that sexual and sex-associated exposures form distinct patterns that are particularly prevalent in this group. Previous research has shown that outbreaks of hepatitis C among HIV-positive men in Europe were linked to MSM-specific viral clusters of HCV [1], [3], [13], [49], **unrelated to injection drug use**, suggesting MSM specific – but not necessarily entirely sexual – transmission modes.

Risk patterns identified by this study encompass **frequent rectal trauma with bleeding**, **frequent engagement in receptive fisting** without gloves, **group sex** and **consumption of nasally administered drugs** (NADs). To our knowledge, this is the first study to specifically collect self-reported data on sex-associated mucosal trauma with subsequent bleeding. We think it is plausible to assume that the amount of *visible* bleeding reported by respondents suggests even higher rates of bleeding that remained unnoticed. Our findings may be corroborated by showing a marked overlap of exposures among cases, e.g. ‘receptive fisting and rectal bleeding’ or ‘history of major surgery and rectal bleeding’. This is consistent with the concept of causal pies in epidemiology, which describes how several causal components can act in concert to produce an effect [50].

Given the strong association of reported surgery with a high number of sex partners, group sex, use of PDE-5 inhibitors (and thus prolonged anal sex), and receptive anal intercourse, we hypothesize that a substantial proportion of the reported surgical interventions had been due to genital or anal condylomata, which are common among HIV-positive gay men with multiple sexual partners [51]–[53]. All types of common proctosurgical interventions for condylomata result in a traumatized anorectal mucosa with lesions and a considerable likelihood of postoperative bleeding. A history of proctosurgery and fast postoperative re-uptake of anal intercourse could explain the overlap of multiple surgical interventions and frequent rectal bleeding.

Our study revealed a considerable proportion of HIV-seroconcordant non-steady sexual partners, contributing to an increased chance of exposure to HCV. Not using condoms with HIV-seroconcordant partners (‘serosorting’) has been described as a risk management approach [54]–[56] that might increase the likelihood of acquiring other STIs than HIV [57]–[61].

HCV retains viability upon environmental exposure to room temperature and drying for at least sixteen hours [62]. This may affect the risk through sharing of equipment for NADs, sharing of sex toys or lubricant, or sharing of sex partners in a group sex setting. Non-injecting recreational **use of amphetamines**, **cocaine**, **or ketamine** is common among gay men with multiple partners, specifically HIV-positive gay men [47], [51], [63]. These substances may be administered orally, rectally or intra-nasally. The equipment for intranasal administration is often shared and may come into contact with mucosal secretions or blood from fellow users. Traumatized nasal mucosa, reactive hyperaemia, and epistaxis have been shown to result from sniffing cocaine powder; blood and HCV were found in nasal secretions of intranasal drug users, and transfer of contaminated blood onto shared sniffing implements has been demonstrated [64]–[66]. A study among *female* non-injecting drug users found that shared implements are likely to serve as vectors for HCV [67]. Given all the above, a rolled bank note that is being circulated at a commercial sex party might be sufficient to expose consumers to HCV-contaminated blood, even without *knowingly* sharing utensils. While it is possible that the association with drug use reflects residual confounding as suggested by others [2], we see no reason to believe that this known transmission route is negligible for gay men.

Our observations are in line with other studies, particularly regarding exposures like **fisting**, **group sex**, **intranasal drug use**, or a combination of these factors [10], [13], [39], [43], [68]. Other research suggests that organized sex parties of predominantly HIV-positive core groups, together with rectal STIs (Shigella, LGV, or rectal gonorrhoea) constitute drivers of sexual HCV transmission. Core groups represent sexual networks characterized by a high number of sex partners who are interconnected [69]. It has been demonstrated that such core groups exist among gay men (In this study nearly all respondents self-identified as gay. As sexual identity is a key factor for forming and sustaining sexual networks, we speak of ‘gay men’ instead of ‘MSM’ [70]). It has further been shown that STIs – particularly rectal STIs with few or no symptoms – accumulate in such groups [57]–[61]. Despite the substantial overlap with consumption of NADs and group sex, **fisting** and **sex-associated rectal bleeding** were not associated with a recent history of bacterial STIs, with a higher number of sex partners, or unprotected anal intercourse. Fisting and sex-associated rectal bleeding might therefore be regarded as additional causal pies, rather than proxy measures for being part of an STI core group, as suggested by other research groups [13].

HCV transmission has often been conceptualized as a pathogen being transferred from an infected insertive man to his receptive partner through his seminal fluid. However the infectivity of semen-derived HCV is being debated; and the presence of HCV RNA in seminal fluid has been demonstrated to be intermittent, and to be independent from blood HCV RNA concentration, immune status, or – in most studies – from HIV co-infection [22], [23], [25], [29], [71]–[75]. Given the strong association with fisting and sex-related rectal bleeding in our study, we suggest that blood rather than semen is the critical medium. An insertive partner's fist (or penis), contaminated with blood, might serve as a vector for subsequent receptive partners in a group sex session, when condoms or gloves are either not applied, or not changed for every new partner – particularly when using a collectively shared supply of lubricant. Lesions in the anorectal mucosa – from fisting, prolonged anal intercourse, or rectal STIs – could serve both as a portal of entry and as a source of infection. Such a vector hypothesis might explain why this and other studies [13], [28] failed to show an independent association of UAI on HCV/HIV co-infection.

### Limitations

A lower than expected response from participating physicians precluded individual matching of study participants. Because of small numbers of cases and controls, the power of the study may not be sufficient to detect less frequent risk factors that may be masked by potential confounders. Despite attempts to minimize recall bias by including only individuals with *acute* hepatitis C and documented seroconversion, some recall bias – especially regarding the time frame ‘since the year 2000’ – cannot be ruled out. Misclassification might have occurred for controls that did not provide dried blood specimens (DBS) and therefore could not be confirmed to be truly seronegative for HCV. However, it is likely that HCV-positives would have been already identified, as German guidelines recommend frequent routine screening of liver enzymes and HCV antibodies among people with HIV [76], as indicated by the high proportion that had been previously screened for HCV antibodies (table 1). Moreover, as a case-cohort design approximates true relative risks in a better way than Odds Ratios in classical case-control studies do [77], [78], this is considered a minor limitation.

It should be underlined that anorectal STIs are profoundly underdiagnosed [79]; hence, the association between hepatitis C and STIs may be underestimated in our study.

While the specific hepatitis C questionnaire addressed the time period prior to seroconversion, the broader KABaSTI questionnaire aimed at the twelve months before study participation, a time frame not exclusively preceding HCV co-infection. It is possible that behaviour or attitudes changed after being diagnosed with and being counselled on acute hepatitis C; this might be particularly applicable to the questions on unprotected anal intercourse or attitudes toward condom use, leading to an underestimation of levels of UAI *before* HCV acquisition among the cases. Given these limitations, and given the fact that all behavioural data is self-reported, causal inferences have to be drawn with caution.

### Conclusions and recommendations

There is a need for specific public health interventions targeting HIV-positive gay men, which address the heightened risk of HCV transmission within their sexual networks. Sexual transmission of HCV seems to occur when HCV-contaminated blood is passed on in a context of situationally-increased HCV prevalence (group sex in HIV-positive gay sexual networks), and mucosal integrity is disrupted due to inflammatory or ulcerative STIs and/or prolonged (PDE-5 inhibitors) or traumatising genito-anal contacts. This risk is heightened in a setting of fisting and sexually-induced anal haemorrhage, particularly following anorectal surgical interventions. If condoms are used but not changed for every new partner, they might work as a vector for blood-borne viruses in a context of sex in a group, regardless of whether the insertive partner is infected with HCV. Similar cross-infection could be facilitated by sharing a glove used during fisting, or a rolled bank note used for consuming NADs. This evidence suggests that public health messages addressing sexual HCV transmission among gay men should focus on the avoidance of sexual and sex-associated exposure to blood rather than seminal fluid. Prevention efforts are needed to communicate sexual or sex-associated routes of transmission for HCV that may not be addressed by messages that focus on the use of condoms.

Proprietors of sex venues need to be educated about HCV transmission risks, and thus to avoid offering shared lubricant supplies. Distributing individual non-traumatising snorting tubes alongside condoms to persons visiting respective sex venues could be considered for harm reduction.

The association of nosocomial risk factors among HIV-positive gay men needs to be elucidated – specifically the interplay of proctosurgery, mucosal disintegrity and the timing of restarting anal intercourse post surgery. Cohort studies on HIV-positive MSM should therefore survey participants' hospital admissions, surgery in general, and proctosurgery in particular.
